# Supplementary material for: Impacts of land use change on native plant-butterfly interaction networks from central Mexico
Source: PeerJ. 2023 Oct 11;11:e16205. doi: 10.7717/peerj.16205 (PMC10576501; doi:10.7717/peerj.16205)
Supplement: Supplemental Information 3 — Values of network metrics for the interaction matrices of different plant-butterfly assemblages in three different habitats, along with the modules identified within each interaction network. [file peerj-11-16205-s003.docx]

**Impacts of land use change on native plant-butterfly interaction networks from central Mexico**

**Table 1S.** Values of network metrics for the interaction matrices of different plant-butterfly assemblages in three different habitats.

| Metric | Native forest | Agricultural | Urban |  |
| --- | --- | --- | --- | --- |
|  |  |  |  |  |
| Number of plant species | 16 | 18 | 44 |  |
| Number of butterfly species | 25 | 21 | 24 |  |
| Number of interactions | 531 | 378 | 524 |  |
| Number of links | 50 | 46 | 83 |  |
| Connectance | 0.12 | 0.12 | 0.07 |  |
| Vulnerability | 7.24 | 3.95 | 2.28 |  |
| Modularity (Q) | 0.54 | 0.56 | 0.61 |  |
| Nestedness (NODF_c) | 2.63 | 1.91 | 2.29 |  |
|  |  |  |  |  |


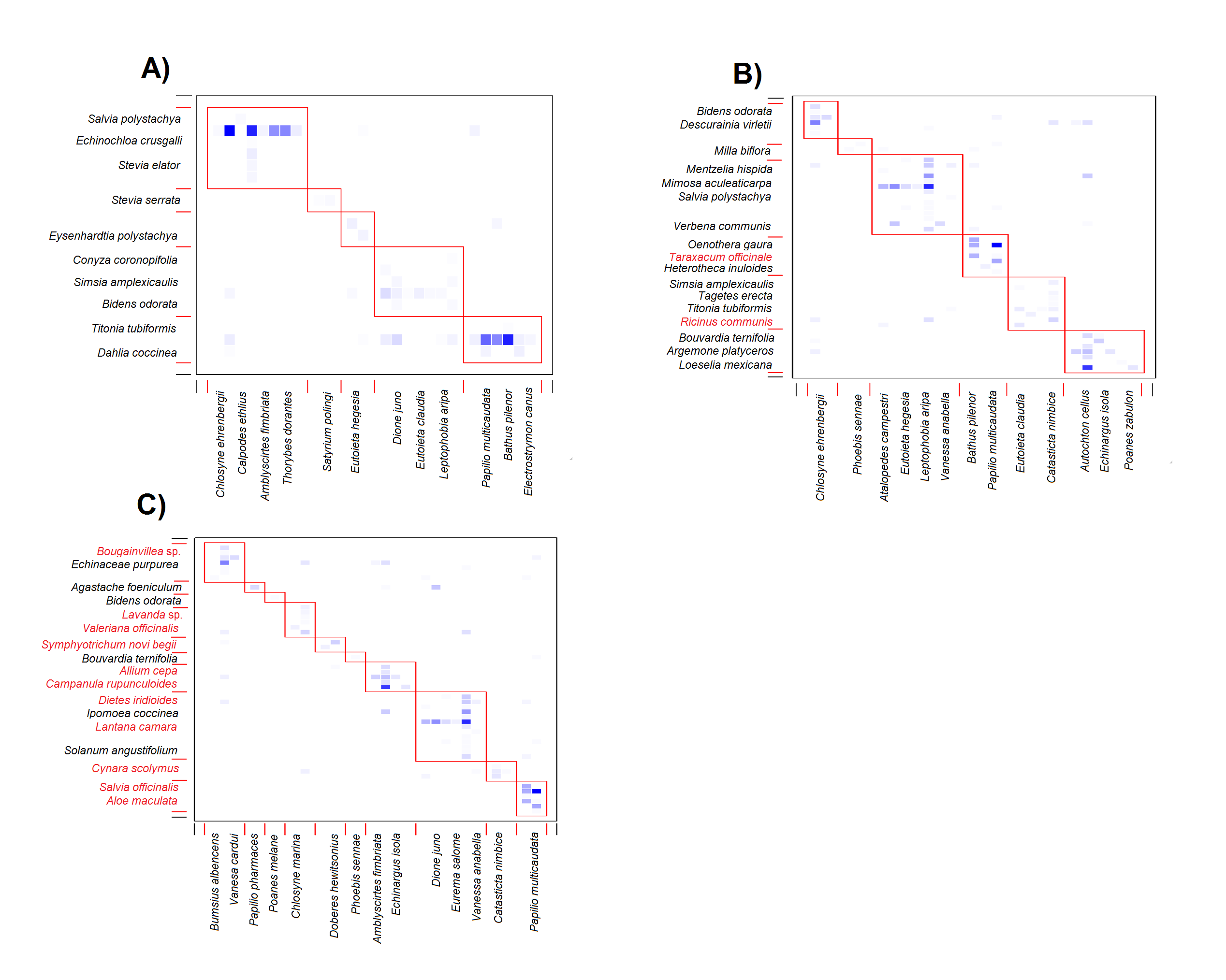


Figure 1S. Plant-butterfly interaction matrix of the A) native forest site, B) agricultural site, and C) urban site, featuring modules identified by QuaBiMo (*Dormann & Strauss, 2014*). Small squares indicate realized interactions, and darker tones indicate more observed interactions. Red square frames delineate modules. To simplify, for each module, only the names of the plant species (vertical axis) and butterfly species (horizontal axis) with the highest number of interactions are shown. Alien plant species are highlighted in red text.
